# Higher risk of malaria transmission outdoors than indoors by *Nyssorhynchus darlingi* in riverine communities in the Peruvian Amazon

**DOI:** 10.1186/s13071-019-3619-0

**Published:** 2019-07-29

**Authors:** Marlon P. Saavedra, Jan E. Conn, Freddy Alava, Gabriel Carrasco-Escobar, Catharine Prussing, Sara A. Bickersmith, Jorge L. Sangama, Carlos Fernandez-Miñope, Mitchel Guzman, Carlos Tong, Carlos Valderrama, Joseph M. Vinetz, Dionicia Gamboa, Marta Moreno

**Affiliations:** 1 Laboratorio ICEMR-Amazonia, Laboratorios de Investigacion y Desarrollo, Facultad de Ciencias y Filosofia, Universidad Peruana Cayetano Heredia, Lima, Peru; 2 Department of Biomedical Sciences, School of Public Health, State University of New York-Albany, Albany, NY USA; 3 Wadsworth Center, New York State Department of Health, Albany, NY USA; 4 Ministry of Health, Iquitos, Peru; 5 Division of Infectious Diseases, Department of Medicine, University of California San Diego, La Jolla, CA USA; 6 Present Address: Section of Infectious Diseases, Yale University School of Medicine, New Haven, CT USA; 7 Departamento de Ciencias Celulares y Moleculares, Facultad de Ciencias y Filosofía, Universidad Peruana Cayetano Heredia, Lima, Peru; 8 Instituto de Medicinal Tropical “Alexander von Humboldt”, Universidad Peruana Cayetano Heredia, Lima, Peru; 9 Present Address: Department of Infection Biology, London School of Hygiene & Tropical Medicine, London, UK

**Keywords:** *Nyssorhynchus darlingi*, Blood meal source, *Plasmodium*, Entomological inoculation rate, Human blood index, GLMM, Mazán District, Peruvian Amazon

## Abstract

**Background:**

Malaria remains an important public health problem in Peru where incidence has been increasing since 2011. Of over 55,000 cases reported in 2017, *Plasmodium vivax* was the predominant species (76%), with *P. falciparum* responsible for the remaining 24%. *Nyssorhynchus darlingi* (previously *Anopheles darlingi*) is the main vector in Amazonian Peru, where hyperendemic *Plasmodium* transmission pockets have been found. Mazán district has pronounced spatial heterogeneity of *P. vivax* malaria. However, little is known about behavior, ecology or seasonal dynamics of *Ny. darlingi* in Mazán. This study aimed to gather baseline information about bionomics of malaria vectors and transmission risk factors in a hyperendemic malaria area of Amazonian Peru.

**Methods:**

To assess vector biology metrics, five surveys (two in the dry and three in the rainy season), including collection of sociodemographic information, were conducted in four communities in 2016–2017 on the Napo (Urco Miraño, URC; Salvador, SAL) and Mazán Rivers (Visto Bueno, VIB; Libertad, LIB). Human-biting rate (HBR), entomological inoculation rate (EIR) and human blood index (HBI) were measured to test the hypothesis of differences in entomological indices of *Ny. darlingi* between watersheds. A generalized linear mixed effect model (GLMM) was constructed to model the relationship between household risk factors and the EIR.

**Results:**

*Nyssorhynchus darlingi* comprised 95% of 7117 Anophelinae collected and its abundance was significantly higher along the Mazán River. The highest EIRs (3.03–4.54) were detected in March and June in URC, LIB and VIB, and significantly more *Ny. darlingi* were infected outdoors than indoors. Multivariate analysis indicated that the EIR was >12 times higher in URC compared with SAL. The HBI ranged from 0.42–0.75; humans were the most common blood source, followed by Galliformes and cows. There were dramatic differences in peak biting time and malaria incidence with similar bednet coverage in the villages.

**Conclusions:**

*Nyssorhynchus darlingi* is the predominant contributor to malaria transmission in the Mazán District, Peru. Malaria risk in these villages is higher in the peridomestic area, with pronounced heterogeneities between and within villages on the Mazán and the Napo Rivers. Spatiotemporal identification and quantification of the prevailing malaria transmission would provide new evidence to orient specific control measures for vulnerable or at high risk populations.

**Electronic supplementary material:**

The online version of this article (10.1186/s13071-019-3619-0) contains supplementary material, which is available to authorized users.

## Background

Malaria endemic riverine communities in Loreto Department, Peruvian Amazon region, are remote and understudied [1–3]. Previous research in this area found that *Nyssorhynchus darlingi* (previously *Anopheles darlingi*, [4]) is the main malaria vector, and often the only Anophelinae species that bites humans [1, 5–7]. However, despite its anthropophilic status, *Ny. darlingi* feeds opportunistically, i.e. we recently reported a high proportion of avian blood meals in three villages to the south and west of Iquitos (Peru) over three years even though the human blood index (HBI) ranged from 0.57 to 0.87 [8]. This finding of high proportions of avian blood meals has implications for local *Plasmodium* transmission. For instance, if access to a human blood meal is blocked by a screen or a net, an avian blood meal can maintain a female *Ny. darlingi* by increasing daily survival, even if a meal on chicken blood is not optimal (at least in the case of *An. gambiae* [9]). If this female were infected with *Plasmodium*, the overall vectorial capacity of the *Ny. darlingi* population could increase slightly if the infected female’s longevity was enhanced by an avian blood meal. Of equal interest, the forage ratio, calculated by a local animal census at the time of mosquito collections, demonstrates a marked preference of *Ny. darlingi* for Galliformes blood compared to human or other available hosts [8]. This pattern has been attributed to both host biomass and availability [8, 10], but its effect on *Plasmodium* transmission remains unclear.

*Nyssorhynchus darlingi* usually rests outdoors (exophilic) and displays heterogeneous feeding behavior throughout its distribution [11–13]. In the Iquitos region, and more broadly, the ratio of outdoor (exophagy) to indoor feeding (endophagy) varies depending on factors such as human behavior, environment and vector control methods [2, 7, 14, 15]. These behaviors influence the entomological indices, human-biting rate (HBR), infectivity rate (IR) and entomological inoculation rate (EIR), that have been a mainstay for comparing the effectiveness of different interventions and estimating malaria risk ([16] but see [17] for novel modifications). Of these indices, the annual EIR is used most commonly to estimate malaria transmission intensity, and for *Ny. darlingi*, the highest EIR recorded is 360.62, in eastern Brazil [18]. In Peru the EIR ranges from 0 to 144 [1, 5, 6, 19]. An annual EIR of 1 was established as a goal for national malaria control programs in sub-Saharan Africa to achieve elimination [20]; more effort needs to be made to obtain such an index in the Neotropics for comparative and monitoring purposes.

In many localities across the Amazon, *Ny. darlingi* is seasonal and its abundance is linked to rainfall and fluctuating river levels [5, 6, 21]. This is most likely associated with the increased numbers and availability of temporary breeding sites suitable for *Ny. darlingi*, such as flooded forest and rainfall pools situated near or alongside lowland rivers [5, 21, 22]. One aspect of ecology that remains unstudied is the potential influence of different river types on diversity, abundance, breeding sites, and perhaps by extension, on *Plasmodium* transmission of Amazonian Anophelinae. Rivers in the Amazon Basin are classified as blackwater, clearwater or whitewater [23]. Generally, blackwater rivers have lower nutrient levels, pH and conductivity compared with whitewater rivers; such physiochemical differences influence the diversity of planktonic flora and fauna. For example, blackwater rivers have been shown to have greater numbers of rotifers but fewer crustaceans, chironomids and mites [24]. Flooded forest is also affected by the nutrient regime of contiguous rivers; thus, the physicochemical attributes of anopheline larval habitats situated on floodplains of whitewater *versus* blackwater rivers are likely to differ. We hypothesized that one potential contributor of seasonal anopheline diversity and abundance is river type. In the present study, we chose to quantify and compare *Ny. darlingi* entomological indices from two villages on the whitewater Napo River and two villages on the blackwater Mazán River. We expected that the *Ny. darlingi* from villages along the whitewater Napo River would demonstrate higher abundance, greater diversity, and higher entomological indices compared with similar villages along the blackwater Mazán River.

Regionally, malaria cases have increased from approximately 11,000 in 2012 to over 55,000 in 2017 [25] with Loreto Department accounting for 96.2% of all recorded cases in Peru. In 2017, *Plasmodium vivax* contributed 76.1% (*n* = 40,120) of the malaria cases followed by *P. falciparum*, which contributed 23.8% (*n* = 12,563) [25]. The main objectives of this study were to gather one year of baseline vector biology data from four hyperendemic malaria communities to determine spatiotemporal local malaria transmission factors, based on river type, blood-feeding habits, *Plasmodium* infectivity and local risk in relation to *Ny. darlingi.*

## Methods

### Collection sites

**Fig. 1:**
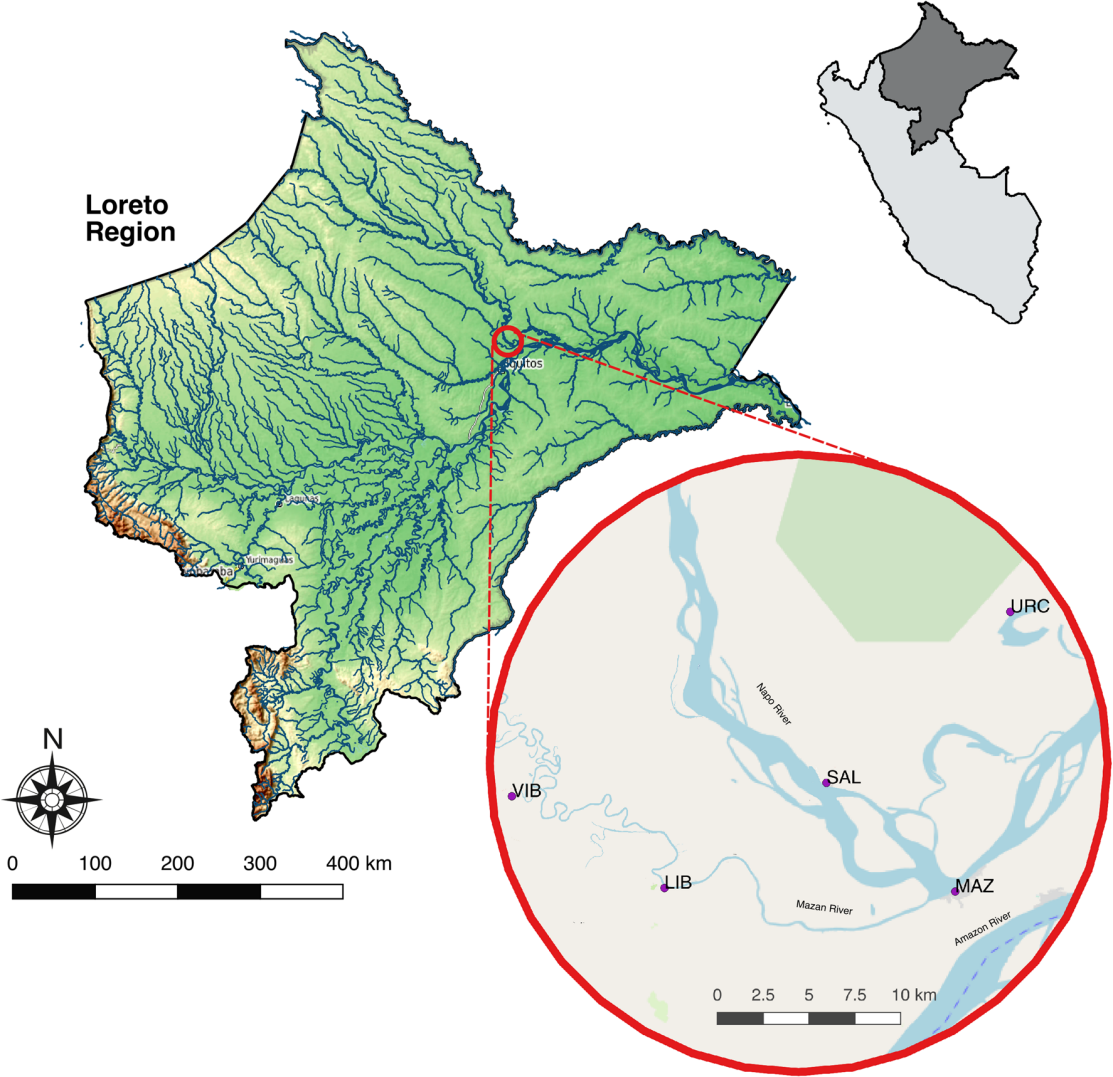
Map showing mosquito collection sites on the Mazán and Napo Rivers, Mazán District, Loreto Department, Amazonian Peru, 2016–2017

Mosquito collections were performed in the Mazán District (Loreto Department, Peru) located 50 km northwest of the city of Iquitos, about 40 minutes by speedboat. Salvador (SAL) and Urco Miraño (URC) are located on the Napo River and Libertad (LIB) and Visto Bueno (VIB) on the Mazán River (Fig. 1). Criteria for choice of communities were based on: (i) annual parasite index (API) > 10; (ii) human population > 50 and < 500; and (iii) location on Mazán or Napo rivers (Table 1). The most common occupations of the inhabitants are fishing, farming and timber extraction. In several Mazán riverine villages, some sector of the human population is mobile [3], seeking work or tending crops away from their village, sometimes for weeks or months at a time [1]. Only Libertad has a health post: residents in Visto Bueno travel to Libertad and those from Urco Miraño and Salvador must travel to Mazán, the largest nearby town (~ 5800 inhabitants), for medical treatment. All four communities are accessible only by boat (distance to Mazán: SAL, 9 km, ~  45 min; URC, 15 km, ~  2 h; LIB, 16 km, ~ 3 h; VIB, 26 km ~ 5 h; time estimated traveling with a 11-horsepower motor boat).

**Table 1:** Overview characteristics of the study sites and malaria cases in Mazán District, Loreto, Peru from 2015 to 2017

Site	River	Coordinates		Population	No. of malaria cases (*Pv*/*Pf*)			API		
		Latitude S	Longitude W		2015	2016	2017	2015	2016	2017
Salvador (SAL)	Napo	3°26′41.6″	73°09′18.4″	390	48 (40/8)	107 (79/28)	27 (15/12)	123	274.3	69.2
Urco Miraño (URC)	Napo	3°21′39.4″	73°03′52.3″	366	139 (135/4)	29 (12/17)	4 (4/0)	379.7	79.2	10.9
Libertad (LIB)	Mazán	3°29′48.7″	73°14′03.8″	245	203 (176/27)	107 (74/33)	144 (112/32)	828.5	436.7	587.7
Visto Bueno (VIB)	Mazán	3°29′48.7″	73°19′01.7″	56	27 (23/4)	22 (12/10)	34 (22/12)	482.1	392.8	607.1

*Abbreviations*: *Pv*, *Plasmodium vivax*; *Pf*, *Plasmodium falciparum*

### Sociodemographic data

A population census for each community was conducted during December 2016–January 2017, with an overall 90% coverage. Each house was identified with a unique number and georeferenced using a handheld global positioning system device (Garmin International Inc., Olathe, KS, USA). In addition, a questionnaire was administered to collect baseline demographic information, health, behavioral and socioeconomic data (i.e. daily net use and coverage household structure and characteristics, travel history, sleeping habits and malaria symptoms) and information was registered on Android tablets. These data were subsequently uploaded into a database that is maintained at the Universidad Peruana Cayetano Heredia in Lima, Peru, and at the School of Public Health, Harvard, USA.

Clinical malaria cases (*Plasmodium vivax* and *P. falciparum*) were documented from malaria episodes by means of passive case detection (with history of fever within the past 24 hours ≥ 38 °C and a positive malaria thick smear after microscopic examination) at local health facilities at the village or district level from the Direccion de Salud de Loreto (DISA-LORETO) in Mazán during the time of the survey (Table 1).

### Mosquito sampling

Five cross-sectional studies were conducted in four communities in 2016–2017 during the dry (2 surveys: June and September 2016) and rainy seasons (3 surveys: March and November 2016, March 2017). Each survey consisted of two nights of human landing catch (HLC) and one night of barrier screens, with a total collection effort of 15 collection nights per village. Indoor and outdoor human landing catch collections were performed (5 m from the main doorway of each house; i.e. peridomestic) for 12 h (18:00–06:00 h), one collector indoors and one collector outdoors working simultaneously, with rotation of personnel every 3 h. Each night two houses, approximately 500 m from each other, were sampled.

To intercept and capture blood-seeking mosquitoes, two barrier screens approximately 15 m long and 2 m high were placed such that the distance from the house/breeding site/resting site was 2–7 m (as described in [8]). This method was performed for one night (18:00–06:00 h) per collection, the night following the first HLC. Resting mosquitoes were sampled by manually searching the surface of the screen with a mouth aspirator every hour, and each side of the screen (facing village/forest and village/river). For both methods, the mosquitoes collected were initially morphologically identified by trained personnel using the available taxonomic keys [26–28]. Mosquitoes were individually stored in microcentrifuge tubes with silica gel in the laboratory in Iquitos at − 20 °C.

To identify and quantify animal hosts (potential sources of blood meals) an animal census was conducted in each community in November 2016 and used to calculate the forage ratio for *Ny. darlingi.* All households were surveyed by consulting residents about presence and number of their domestic animals (dog, cat, chicken, turkey, pig, cow and duck) and observation of any feral animals (Additional file 1: Table S1).

### Laboratory procedures

A subsample of specimens from HLC and barrier screen collections that could not be identified morphologically (cryptic *Nyssorhynchus* species, damaged specimens, etc.) was identified using ITS2-PCR-RFLP [29], or by sequencing the BOLD region of *cox*1 [30] and querying against the BOLD Identification System (http://www.boldsystems.org) or GenBank (https://www.ncbi.nlm.nih.gov/genbank). To detect the source of a blood meal, total genomic DNA of each mosquito specimen was extracted from the abdomen using the DNeasy Blood & Tissue Kit (Qiagen, Hilden, Germany). *Cytochrome b* PCR-RFLP assays were performed to identify mammalian blood meal hosts (human, cow, pig and dog) [31]. In addition, Galliformes [32], rat and monkey hosts were included in the analysis [33, 34]. Results were visualized in agarose gel and no sequencing was performed for any sample.

Heads and thoraces from specimens were analyzed in pools of 3–6 individuals (same collection site/time) to detect *P. vivax* (PV247-PV210) and *P. falciparum* using an enzyme-linked immunosorbent assay (ELISA) [35]. ELISA kits (MR-1028K) were obtained from BEI Resources, NIAID, NIH and *Plasmodium vivax* Sporozoite ELISA Reagent Kit, MRA-1028K, contributed by Robert A. Wirtz. Female *Ny. darlingi* (colony specimens) were used as negative controls and female *Ny. darlingi* infected with *P. vivax* as positive controls. Optical density was measured at 410 nm in a Bio-Rad ELISA plate reader (Bio-Rad, Hercules, CA, USA) 60 min after addition of the substrate. The cut-off for positive samples was determined by multiplying the average OD (negative controls) by twice (2×) the negative control for each plate. Fifty-five mosquito samples, that were at or near the limit of detectability, were retested individually by real-time PCR following the protocol in Bickersmith et al. [36].

### Data analysis

#### Linking human census and malaria case data

Clinical malaria records from the passive case detection of the Ministry of Health (MoH) were linked with the study dataset to identify malaria episodes in our study participants. The patients’ names were the only variable shared between the study database and the malaria notification system. Since names may have been misspelled and incorrectly entered, a normalized extension of the Generalized Levenshtein Distance was used [37, 38]. This is defined as the minimum cost of transforming one string into another through a sequence of weighted edit operations following the equation

$$ {\text{d}}_{{{\text{N}} - {\text{GLD}}}} \left( {{\text{X}},{\text{Y}}} \right) = \frac{{2 \cdot {\text{GLD}}\left( {{\text{X}},{\text{Y}}} \right)}}{{\left( {\left| {\text{X}} \right| + \left| {\text{Y}} \right|} \right) + {\text{GLD}}\left( {{\text{X}},{\text{Y}}} \right)}} $$

An entry from the malaria notification system was associated with an individual from the census database if both have names with normalized GLD less or equal than 0.1. Moreover, to include multiple misspellings on the malaria notification system database, all entries were associated with the same individual if they have the same normalized GLD.

#### Entomological indicators

Human-biting rate (HBR) was calculated as the average number of *Ny. darlingi* bites per collector per hour. Infection rate (IR) is the proportion of *Ny. darlingi* that was determined to be *Plasmodium* positive. Parity was measured in only 40 specimens and the sample size was too small for results to be meaningfully included. These 40 individuals were used in the calculation of HBR but not tested for *Plasmodium* and not used to calculate IR/EIR. For calculation of entomological indices, specimens of mixed infections with *P. vivax* and *P. falciparum* were added to totals of each of these parasites [39]. The entomological inoculation rate (EIR) was calculated by multiplying the HBR (monthly) and IR. These indices were calculated for each collection (month) for each of the four communities. The human blood index (HBI) was calculated as the proportion of mosquitoes fed on a specific host divided by the number of mosquitoes analyzed (mixed blood meals were added to totals of each host) as in [8]. To test for significance between human and non-human blood meals in *Ny. darlingi* among seasons and localities we employed Chi-square analysis. To quantify blood meal source, host data recorded in the census were used to calculate the forage ratio (*w*_i_) [40, 41] and selection index (*B*_*i*_) [42]. The weight of each blood meal source was determined following Moreno et al. [8]. We employed the R bipartite package [43] to generate a host vector quantitative interaction network for the four communities as in Moreno et al. [8].

#### Statistical analysis

Fisher’s exact test was used for significance testing of categorical factors for each *Plasmodium* spp. To assess significant differences in EIR, a generalized linear mixed effect model (GLMM) was used to handle the nested structure of sampled data: two locations (intra- and peridomestic) sampled per survey, nested within 16 households. A GLMM assuming a negative binomial distribution for the error term, and a log link function was constructed to model the relationship between household risk factors and the EIR. We estimated an incidence rate ratio (IRR) for the association between EIR and intra- and peridomestic collection, study area, number of inhabitants per household, inhabitants/room ratio, whether family was recently settled in the community, number of bednets per household, years since last bednet impregnation, and electricity supply. Statistical significance was defined as a *P*-value < 0.05 and 95% confidence intervals (CI) were estimated as appropriate. Factors with *P*-values < 0.2 for the Wald test in the univariate analysis were included in the multivariate model. Using a backward stepwise process, the final model retained all factors that were significantly associated with EIR. Interactions were systematically checked for up to order two. Likelihood ratio tests (LRTs) were used to assess statistical differences between nested models. All data analyses were conducted in STATA v.15.1 (StataCorp, College Station, TX, USA).

## Results

### Characteristics of the study sites and association with malaria cases

From December 2016 to January 2017 a census was conducted in the four sites of the survey with a coverage of 90% across the study sites. Over 96% of the households in the communities have some kind of bednet, either purchased locally or distributed in 2016 by DIRESA-LORETO (long-lasting insecticide nets, LLINs) (Table 2). Malaria cases reported differed by study site for both *P. vivax* and *P. falciparum*, with the highest case numbers in SAL and LIB. Occupation as a housewife or student and having electricity were associated only with *P. vivax* malaria (*P* = 0.003 and *P* = 0.103, respectively) whilst pona (palm *Iriartea deltoidea*, Family *Arecaceae*) *vs* wood wall material was associated with *P. falciparum* (*P *= 0.034). Neither *P. vivax* nor *P. falciparum* infection was associated with gender or age, education level, dwelling roof material, or ownership of bednets (LLINs and non-treated) (Table 3).

**Table 2:** Bednet coverage by site. Data from population census in December 2016 to January 2017

Site	River	Only Tocuyo net^a^	At least one LLIN	Total	No. of households
Salvador (SAL)	Napo	9 (13.4%)	58 (86.6%)	100%	67
Urco Miraño (URC)	Napo	9 (18.7%)	39 (81.3%)	100%	48
Libertad (LIB)	Mazán	10 (18.5%)	42 (77.8%)	96.3%	54
Visto Bueno (VIB)	Mazán	3 (20.0%)	12 (80.0%)	100%	15

^a^Non-impregnated bednets locally made of woven cotton fabric

**Table 3:** Baseline characteristics of the study population and its association with *P. vivax* and *P. falciparum* malaria cases

Characteristics	*P. vivax*			*P. falciparum*		
	No. positive (%) (*n* = 42)	No. negative (%) (*n* = 808)	*P*-value	No. positive (%) (*n* = 35)	No. negative (%) (*n* = 878)	*P*-value
Study area			0.001**			0.008**
Salvador	20 (47.6)	282 (34.9)		11 (61.1)	291 (35.0)	
Urco Miraño	3 (7.1)	246 (30.4)		0 (0.0)	249 (29.9)	
Libertad	18 (42.9)	227 (28.1)		6 (33.3)	239 (28.7)	
Visto Bueno	1 (2.4)	53 (6.6)		1 (5.6)	53 (6.4)	
Sex			0.636			0.815
Female	22 (52.4)	388 (48.0)		8 (44.4)	402 (48.3)	
Male	20 (47.6)	420 (52.0)		10 (55.6)	430 (51.7)	
Age group (years)			0.781			0.456
<10	15 (35.7)	259 (32.0)		4 (22.2)	270 (32.5)	
10–29.9	11 (26.2)	255 (31.6)		8 (44.4)	258 (31.0)	
≥30	16 (38.1)	294 (36.4)		6 (33.3)	304 (36.5)	
Education			0.343			0.682
None	11 (26.2)	223 (27.6)		4 (22.2)	230 (27.6)	
Primary school	27 (64.3)	440 (54.5)		12 (66.7)	455 (54.7)	
Secondary school or higher	4 (9.5)	145 (17.9)		2 (11.1)	147 (17.7)	
Occupation (>18 years-old)			0.003**			0.254
Forest related	5 (11.9)	260 (32.2)		9 (50.0)	256 (30.7)	
Housewife/student	32 (76.2)	409 (50.6)		7 (38.9)	434 (52.2)	
Others	5 (11.9)	139 (17.2)		2 (11.1)	142 (17.1)	
Dwelling wall material^a^			1.000			0.034**
Wood	35 (92.1)	630 (92.4)		13 (76.5)	652 (92.7)	
Pona and other related	3 (7.9)	52 (7.6)		4 (23..5)	51 (7.3)	
Dwelling roof material			0.745			0.321
Tin (*calamina*)	14 (33.3)	293 (36.3)		4 (22.2)	303 (36.4)	
Palm leaves	28 (66.7)	515 (63.7)		14 (77.8)	529 (63.6)	
Electricity			0.103*			0.276
Yes	16 (38.1)	207 (25.6)		11 (61.1)	616 (74.0)	
No	26 (61.9)	601 (74.4)		7 (38.9)	216 (26.0)	
Impregnated bednets			0.381			0.336
Yes	38 (90.5)	680 (84.2)		17 (94.4)	701 (84.3)	
No	4 (9.5)	128 (15.8)		1 (5.6)	131 (15.7)	

*P < 0.2, **P < 0.05; Fisher’s exact test

^a^Factor with missing values

### Species composition and biting behavior

Overall, for both HLC and barrier screen methods, 7117 female Anophelinae specimens were collected in the five surveys in the four sites (Table 4). By HLC, 6365 Anophelinae were captured: VIB (*n* = 3530); Libertad (*n* = 2083); Urco Miraño (*n* = 679); and Salvador (*n* = 73). *Nyssorhynchus darlingi* Root, 1926, was the most abundant species in each of the four communities (99% in Visto Bueno; 96% in Libertad; 78% in Urco Miraño; and 67% in Salvador). Other Anophelinae species identified were *Ny. nuneztovari* Gabaldon, 1940 (*s.l.*), *Ny. oswaldoi* Peryassú, 1922 (*s.l.*), *Ny. benarrochi* Gabaldon, Cova-Garcia & Lopez, 1941 B, *Ny. rangeli* Gabaldon Cova-Garcia & Lopez, 1940, *Ny. dunhami* Causey, 1945, *Ny. triannulatus* Neiva & Pinto, 1922, *Ny.* sp. nr. *konderi*, *An*. *mattogrossensis* Lutz & Neiva, 1911, *An. forattinii* Wilkerson & Sallum, 1999, *An. costai* Fonseca & Silva Ramos, 1939, *An. forattinii*/*An. costai*, *An.* sp. nr. *forattinii*, and several specimens identified only to genus level (Table 4). In general, species richness was lower for HLC than barrier screen in all communities, and it was near-equal for the Mazán River communities (*n* = 8) vs those along the Napo River (*n* = 10).

**Table 4:** *Nyssorhynchus* and *Anopheles* species identification and composition in SAL, URC, LIB, and VIB communities, Mazán District, Peru in 2016–2017 by HLC and barrier screen mosquito collection methods

Site	Year	Month	Method	
			HLC (*n*)	Barrier screen (*n*)
SAL	2016	March	*Ny. darlingi* (2)	
			*Nyssorhynchus* spp. (2)	
			*An. forattinii*/*An. costai* (1)	
				*Anopheles* spp. (1)
		June	*Ny. darlingi* (60)	*Ny. darlingi* (10)
			*Ny. nuneztovari* (*s.l.*) (1)	
			*Nyssorhynchus* spp. (4)	*Nyssorhynchus* spp. (14)
			*forattinii*/*An. costai* (1)	
				*Anopheles* spp. (7)
				*Ny. benarrochi B* (2)
		September	*Ny. darlingi* (2)	
				*Nyssorhynchus* spp. (3)
		Total (*n*)	73	37
URC	2016	March	*Ny. darlingi* (15)	
			*Ny. oswaldoi* (*s.l.*) (1)	
			*An*. *forattinii*/*An. costai* (1)	
		June	*Ny. darlingi* (487)	*Ny. darlingi* (133)
			*An. forattinii*/*An. costai* (3)	
			*An.* sp. nr. *forattinii* (1)	*An.* sp. nr. *forattinii* (1)
			*An. mattogrossensis* (1)	
			*Nyssorhynchus* spp. (133)	*Nyssorhynchus* spp. (24)
				*Anopheles* spp. (8)
				*Ny. triannulatus* (*s.l.*) (1)
				*Ny. rangeli* (1)
				*Ny. dunhami* (2)
				*Ny*. sp. nr. *konderi* (1)
		September	*Ny. darlingi* (18)	
			*Nyssorhynchus* spp. (1)	
	2017	February	*Ny. darlingi* (15)	*Ny. darlingi* (1)
			*Nyssorhynchus* spp. (1)	*Nyssorhynchus* spp. (1)
			*Anopheles* spp. (2)	
		Total (*n*)	679	173
LIB	2016	March	*Ny. darlingi* (188)	*Ny. darlingi* (8)
			*Ny. dunhami* (1)	
			*Ny. oswaldoi* (*s.l.*) (1)	
			*An.* sp. nr. *forattinii* (4)	
			*An. forattinii*/*An. costai* (1)	
			*Nyssorhynchus* spp. (74)	
		June	*Ny. darlingi* (1439)	*Ny. darlingi* (101)
			*An. forattinii*/*An. costai* (2)	
			*An. mattogrossensis* (1)	
				*Anopheles* spp. (1)
				*Nyssorhynchus* spp. (4)
		September	*Ny. darlingi* (72)	*Ny. darlingi* (3)
			*Nyssorhynchus* spp. (1)	
		November	*Ny. darlingi* (4)	
	2017	February	*Ny. darlingi* (294)	
			*Nyssorhynchus* spp. (1)	
		Total (*n*)	2083	117
VIB	2016	March	*Ny. darlingi* (370)	*Ny. darlingi* (4)
			*Ny. oswaldoi* (*s.l.*) (2)	
			*Ny. benarrochi* B (1)	
			*Nyssorhynchus* spp. (13)	
			*forattinii*/*An. costai* (2)	
			*An*. sp. nr. *forattini* (1)	
				*Anopheles* spp. (1)
		June	*Ny. darlingi* (2730)	*Ny. darlingi* (406)
		September	*Ny. darlingi* (141)	*Ny. darlingi* (12)
			*Nyssorhynchus* spp. (1)	*Nyssorhynchus* spp. (1)
		November	*Ny. darlingi* (3)	
			*forattinii* (1)	
	2017	February	*Ny. darlingi* (264)	*Ny. darlingi* (1)
			*Nyssorhynchus* spp. (1)	
		Total (*n*)	3530	425

**Fig. 2:**
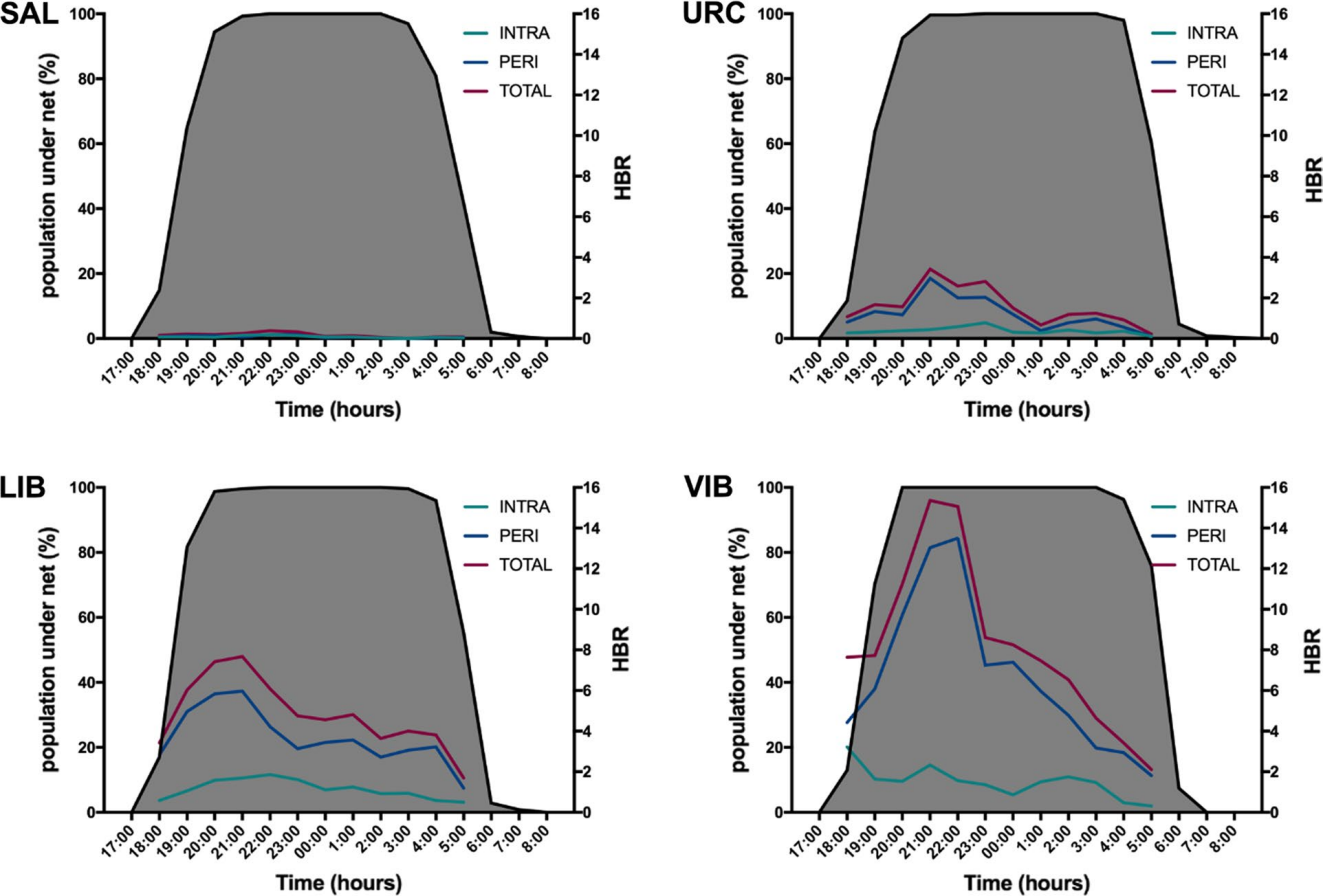
Mean indoor (intradomestic) and outdoor (peridomestic) human-biting rate at four communities in the Mazán District 2016–2017. **a** Salvador (SAL). **b** Urco Miraño (URC). **c** Libertad (LIB). **d** Visto Bueno (VIB). No specimens were collected in November and March 2017 in Salvador or in November 2016 in Urco Miraño. Right Y-axis is the human-biting rate. Grey background shading represents the proportion of the human population under bednets (left Y-axis) each hour of the night (X-axis)

There were more Anophelinae collected consistently in the peridomestic area (*n* = 5024; 78.9%) compared with inside houses (*n* = 1341; 21.1%) in all communities, and more biting occurred prior to midnight (Fig. 2 and Additional file 1: Table S2), with 56% of all mosquitoes collected from 18:00 to 22:00 h. In SAL there were two outdoor peaks and one indoors before 23:00 h. In URC, the peak biting time was earlier outdoors (21:00 h) than indoors (23:00 h). In LIB, there were no real biting peaks and in VIB most outdoor biting occurred between 21:00 and 23:00 h and indoor biting was uninterrupted until 3:00 h.

### *Plasmodium* detection in mosquitoes and entomological indices

All *Ny. darlingi* samples collected by HLC were tested in pools of 3–5 specimens using ELISA, and calculations were based on the conservative assumption of *n* = 1 infected specimen per pool. The overall infection rate (IR) was 1.6% (91/5870 specimens of *Ny. darlingi* tested) and this varied among communities: Libertad, 27/1895 (1.4% IR); Visto Bueno, 50/3394 (1.5% IR), Urco Miraño, 13/519 (2.5% IR); and Salvador, 1/62 (1.6% IR) were positive (Table 5). *Plasmodium vivax* V210 was the most frequent variant in 62 pools (SAL = 0, URC = 6, LIB = 12, VIB = 44), followed by *P. falciparum* in 19 pools (SAL = 0, URC = 2, LIB = 12, VIB = 5) and *P. vivax* V247 in 10 pools (SAL = 1, URC = 5, LIB = 3, VIB = 1). Seven pools included specimens with mixed infections.

**Table 5:** Infection rate (IR), human-biting rate (HBR) and entomological inoculation rate (EIR) by month in four Mazán District communities between 2016 and 2017; Napo River (SAL and URC) and Mazán River (LIB and VIB)

Collection date	IR		HBR		EIR		IR		HBR		EIR	
	In	Out	In ± SE	Out ± SE	In	Out	In	Out	In ± SE	Out ± SE	In	Out
**SAL**							**URC**					
Mar 2016	0	0	0.3 ± 0.3	1.0 ± 1.0	0	0	0	0	1.0 ± 1.3	3.0 ± 2.0	0	0
Jun 2016	0.03	0	8.3 ± 5.8	8.3 ± 3.8	0.3	0	0.01	0.03	37.3 ± 24.3	119 ± 37.5	0.3	3.60
Sep 2016	0	0	0.5 ± 0.5	0	0	0	0	0	0.8 ± 0.3	4.0 ± 0	0	0
Nov 2016	0	0	0	0	0	0	0	0	0	0	0	0
Mar 2017	0	0	0	0	0	0	0.14	0	1.8 ± 0.8	2.8 ± 0.3	0.3	0
**LIB**							**VIB**					
Mar 2016	0.02	0.02	34.0 ± 26.0	33.3 ± 4.7	0.8	0.72	0.02	0.07	29.8 ± 3.3	67.5 ± 6.0	0.58	4.54
Jun 2016	0.03	0.01	65.8 ± 9.25	294.8 ± 107.8	2.3	3.52	0.03	0.01	114.3 ± 14.8	568.3 ± 12.8	3.05	3.03
Sep 2016	0.00	0.02	4.3 ± 1.3	14.0 ± 2.0	0	0.34	0	0.04	4.5 ± 1	31.0 ± 14.5	0	1.19
Nov 2016	0.00	0.25	0	1.0 ± 1.0	0	0.25	0	0	0.3 ± 0.3	0.8 ± 0.7	0	0
Mar 2017	0.00	0	19.3 ± 11.3	54.5 ± 14.0	0	0.00	0.02	0.01	13.3 ± 4.8	53 ± 8.5	0.28	0.29

*Abbreviation*: SE, standard error

HBR, calculated only for *Ny. darlingi*, presented a similar trend in all communities and peaked in June 2016 (Table 5). In Visto Bueno, HBR ranged from 0.3 to 568.3 b/p/n; in Libertad, from 0 to 294.8 b/p/n; in Urco Miraño, from 0 to 119 b/p/n; and in Salvador, from 0 to 8.3 b/p/n. The distribution of infected mosquitoes across the 12 h collections varied by month and locality. More were captured before 24:00 h, i.e. 55/91. The highest EIRs were detected in March and June, mostly outdoors (Table 5). According to the 2016 census, bednet use was ~100% and most community inhabitants were virtually covered between 19:00 and 4:00 h (with either LLINs or untreated bednets). Bednet coverage was very high and similar in all communities (Table 2, Fig. 2).

### Risk factors for malaria transmission

Among the study sites, the multivariate analysis indicated that the EIR was >12 times higher in URC (IRR: 12.49; 95% CI: 0.65–238.1; *P* = 0.093), > 26 in LIB (IRR: 26.61; 95% CI: 1.47–480.6, *P* = 0.026) and > 56 times higher in VIB (IRR: 56.46; 95% CI: 3.19–997.8; *P* = 0.006) compared with SAL (Table 6). The EIR was >2-fold higher in the peridomestic *vs* intradomestic area in both uni- (IRR: 2.42; 95% CI: 1.23–4.76; *P* = 0.010) and multivariate models (IRR: 2.46; 95% CI: 1.23–4.89; *P* = 0.010). Recent settlement in the community, electricity and time of the last bednet insecticide impregnation were associated with a higher risk of EIR but these factors were not retained in the multivariate model (Table 6).

**Table 6:** Fixed effects of univariate and multivariate multilevel negative binomial models of the entomological inoculation rate (EIR). Data analysis is based on census in December 2016–January 2017 and entomological data incorporated from the 5 surveys starting March 2016 (12 month period)

	Univariate			Multivariate		
	IRR	95% CI	*P*-value	IRR	95% CI	*P*-value
Null model						<0.001
Constant	0.08	0.02–0.33	<0.001	0.003	0.0001–0.06	
Study area (ref = Salvador)						
Libertad	26.50	1.46–479.4	0.027**	26.61	1.47–480.6	0.026**
Urco Miraño	12.62	0.66–240.8	0.092*	12.49	0.65–238.1	0.093*
Visto Bueno	56.32	3.18–997.1	0.006**	56.46	3.19–997.8	0.006**
Location (ref = intradomestic)						
Peridomestic	2.42	1.23–4.76	0.010**	2.46	1.23–4.89	0.010**
No. of inhabitants per household	0.91	0.72–1.16	0.455			
Inhabitants/room ratio	0.89	0.67–1.17	0.398			
Recent settlement in community (ref = no)						
Yes	0.26	0.04–1.81	0.172*			
No. of bednets	0.95	0.64–1.39	0.785			
Years since last bednet impregnation (ref = never)						
One year	0.32	0.10–1.10	0.07*			
Two years	0.45	0.12–1.68	0.233			
Electricity (ref = no)						
Yes	4.66	1.93–11.24	0.001**			

Mixed-effects negative binomial models, with random intercepts, Wald Test *P*-value, *P < 0.2, **P < 0.05

*Abbreviations*: IRR, incidence rate ratio; CI, confidence interval

### Blood meal source identification

Eight different species of Anophelinae were collected on the barrier screens (*n* = 752) (Table 4). *Nyssorhynchus darlingi* (*n* = 679) was the most abundant, followed by *Ny. dunhami* (*n* = 2), *Ny. benarrochi* B (*n* = 2), *Ny. rangeli* (*n* = 1), *An.* sp. nr. *forattinii* (*n* = 1), *Ny. triannulatus* (*s.l*.) (*n* = 1), *Ny. konderi* (*n* = 1), *Anopheles* (*Anopheles*) spp. (*n* = 18) and several damaged specimens that could not be identified (*n* = 47) (Table 4). Nearly all mosquitoes (95%) examined during this study were captured in June 2016 (716/752); the lowest monthly capture was November 2016. More Anophelinae (*n* = 415) were captured on the village-forest barrier screen compared with the village-breeding barrier screen (*n* = 337). Because mosquito abundance in June accounted for > 95% of all the mosquitoes collected, data analyses comparing abundance among months or seasons were not performed. Regarding screen side, 41.2% of the mosquitoes were captured on the village side, 29.7% on the forest side and 28.9% on the breeding/river side (Additional file 1: Figure S1). Abundance was highest from 21:00 to 24:00 h (*n* = 344), and then evenly distributed among the remaining three time periods: 18:00–21:00 h (*n* = 184); 24:00–3:00 h (*n* = 128) and 3:00–6:00 h (*n* = 96). Only 6.8% of the Anophelinae were determined by visual inspection to be blood-fed.

**Fig. 3:**
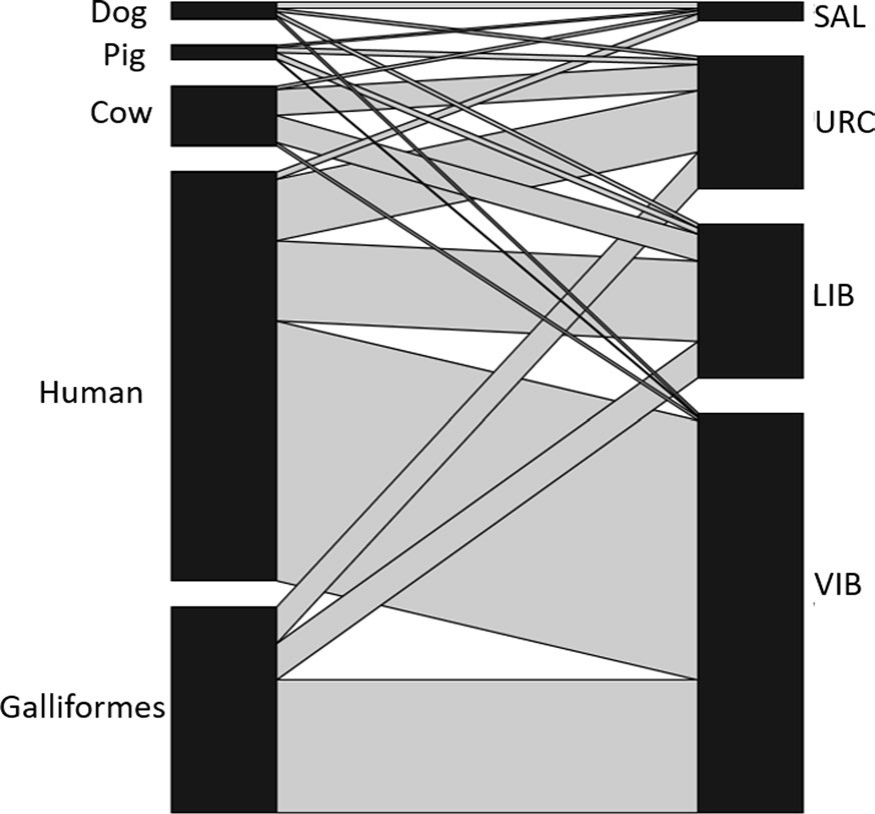
Quantitative interaction network of *Ny*. *darlingi* blood meal sources in SAL, URC, LIB and VIB, 2016–2017. Network is constructed based on blood meal source analysis for 622 *Ny*. *darlingi* females

**Fig. 4:**
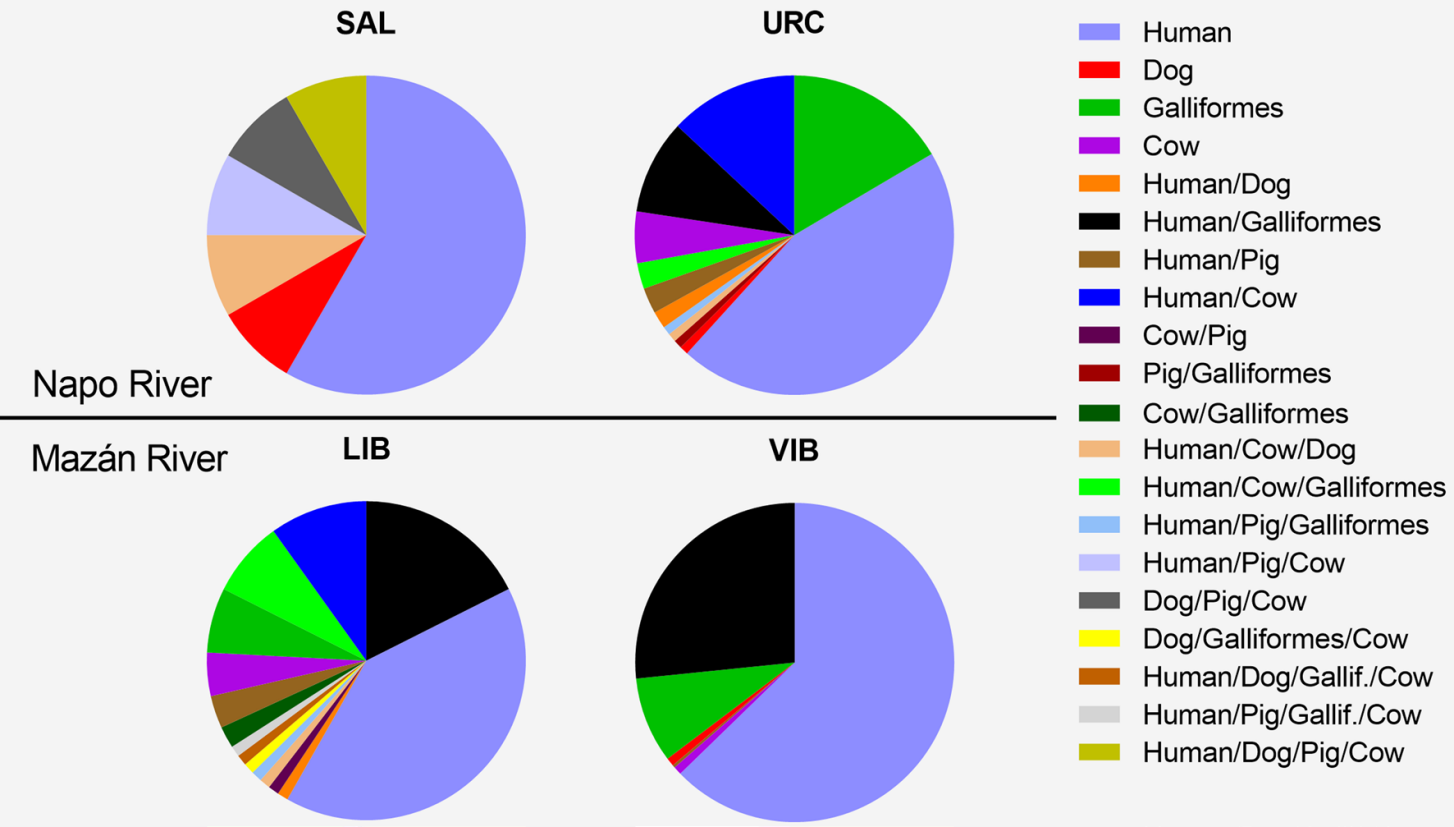
Proportion of blood meal sources based on *Ny. darlingi* collected by barrier screens in Mazán District between 2016 and 2017

Blood meal source was determined for 699 *Ny. darlingi* as follows: VIB (*n* = 425); URC (*n* = 143); LIB (*n* = 107); and SAL (*n* = 24) (Table 7). Single-host blood meals accounted for the highest percentage of those identified (68.5%; 394/575) and humans were the most common source of blood (81%; 320/394). This was followed by Galliformes (14.2%; 56/394), cows (3.3%; 13/394) and dogs (1.2%; 5/394); blood meal source could not be identified for 18% of the samples. Multiple blood meals were found in 181 mosquitoes (31.5%). Double feeds (*n* = 160) were distributed among three communities: (VIB = 96, LIB = 32 and URC = 32). Triple feeds were found in LIB (*n* = 10), URC (*n* = 5) and SAL (*n* = 3), and quadruple feeds (*n* = 3) only in LIB and SAL (Table 7). There were significant differences in the proportion of human blood meals among the four sites (*χ*^2^ = 23.9, *df* = 3, *P* < 0.0001), with a higher proportion of human blood meals in VIB than in the other three sites (0.75 in VIB, 0.72 in LIB, 0.62 in URC and 0.42 in SAL). Forage ratio A (based on number of animals) was highest for humans only in VIB. In URC and LIB, it was highest for cows and in SAL for pigs. In contrast, forage ratio B (based on biomass calculated by multiplying the estimated weight of each animal by its abundance according to the animal census) was highest for Galliformes in URC, LIB and VIB, and for cows in SAL (Additional file 1: Table S3). The quantitative interaction network of blood meal source by locality (Fig. 3) supported patterns of organization based on the trophic preference (human, chicken, cow, pig and dog) from the four mosquito populations (SAL, URC, LIB and VIB). Figure 4 shows the proportion of all single and multiple blood meals in each community.

**Table 7:** Summary of blood meal sources identified in the abdomen of *Nyssorhynchus darlingi* collected by barrier screens

Host blood meal	Site			
	SAL	URC	LIB	VIB
Single blood meals				
Human	7	52	37	224
Dog	1	1	–	3
Galliformes	–	19	6	31
Cow	–	6	4	3
Total	8	78	47	261
Mixed blood meals				
Human/dog	–	2	1	–
Human/Galliformes	–	11	16	95
Human/pig	–	3	3	1
Human/cow	–	15	9	–
Cow/pig	–	–	1	–
Pig/Galliformes	–	1	–	–
Cow/Galliformes	–	–	2	–
Human/cow/dog	1	1	1	–
Human/cow/Galliformes	–	3	7	–
Human/cow/pig	1	–	–	–
Human/pig/Galliformes	–	1	1	–
Dog/pig/cow	1	–	–	–
Dog/Galliformes/cow	–	–	1	–
Human/dog/Galliformes/cow	–	–	1	–
Human/pig/Galliformes/cow	–	–	1	–
Human/dog/pig/cow	1	–	–	–
Total	4	37	44	96
Not identified	12	28	16	68
Total	24	143	107	425

Based on the present study, we found no support for the hypothesis that there would be greater abundance, higher species richness, and higher entomological indices in the communities along the whitewater Napo River (SAL, URC) compared with the blackwater Mazán (LIB, VIB). In fact, the trends were nearly opposite: abundance and entomological indices were higher in the Mazán River communities, and species richness was nearly identical for Anophelinae along the two river systems. There are many environmental variables that influence these aspects of the vector biology of *Ny. darlingi*, but either river type is not one of these, or we did not sample an adequate number of populations, or not for long enough, to determine any effect.

## Discussion

Entomological surveillance is imperative to characterize malaria transmission patterns that will lead to planning adequate vector control measures in a specific context. Overall, this study reflects the behavioral heterogeneity of *Ny. darlingi* for biting pattern, location and feeding choices at a microgeographical scale. Remarkably, our findings show that EIR is significantly higher in the peridomestic area (exophagic mosquitoes) in all study sites, but particularly elevated in the Mazán River micro-basin. Contrary to our hypothesis, our study shows that the present study sites (villages) located on the bank of blackwater rivers have higher mosquito abundance and EIR than those on whitewater rivers. Characterization of the biotic and abiotic factors that determine the distribution of Anophelinae larval habitats in both watersheds will provide a more comprehensive malaria transmission scenario. For instance, Prussing et al. [44] have outlined some components correlated with aquatic larval habitats of *Ny. darlingi* in the peri-Iquitos area, such as low forest coverage, low sunlight exposure and emergent vegetation.

Our findings support those of a prior study that revealed *Ny. darlingi* to be the predominant Anophelinae species in the Mazán and Napo river communities [1]. The abundance of this species was higher along the Mazán River compared with the Napo River regardless of seasonality. More specifically, the HBR in Visto Bueno was two-fold higher than in Libertad (same river), and 4-fold and 68-fold higher than in Urco Miraño and Salvador, respectively, both on the Napo River. The HBR varied by season, with the highest value detected in June in all localities. This contrasts with the *Ny. darlingi* peak in other communities in peri-Iquitos, for example in Lupuna, Cahuide and Villa del Buen Pastor, where the highest HBR was reported in March-April for the same mosquito species [6]. Because mosquito abundance in this ecological scenario is more related to river levels than to precipitation [5–7], differences may be explained due to the different watersheds under observation [i.e. Mazán and Napo rivers in the present study compared to the Itaya and Nanay rivers (closer to Iquitos) in the above-mentioned studies].

In general, *Ny. darlingi* prefers to bite outdoors independently of the site, although the outdoor:indoor ratio varies slightly and it decreases in March, probably when mosquitoes are seeking shelter during the rainy season [7, 13] or due to differences in microclimatic conditions inside the houses [45–47]. Outdoor feeding behavior has been described commonly in *Ny. darlingi* in the region [5, 6, 48], but a recent trend of increased feeding inside houses has been reported [7] possibly because of a combination of efficacy-loss of LLINs distributed by the government and a reduction of IRS for the last five years in the region. In the present study sites, bednet coverage was high (over 77% of the population covered by LLINs) and a high proportion of individuals would be protected when using them during the night. Salvador was the only site with a similar proportion of mosquitoes biting inside and outside the houses, although mosquito sample size was very low. We acknowledge that bednet ownership and population coverage data variables are not sufficient to estimate the accessibility of mosquitoes to humans during the night time and other sociodemographic characteristics such as bednet use, vulnerable population sleeping under a bednet (children under five years-old) and malaria knowledge should be integrated in a more comprehensive study [49]. For instance, in an observational study in the rural communities of the Peruvian Amazon [50], nets were lifted a mean of 6.1 times per night. The authors conclude that this bednet use pattern may contribute to residual transmission.

The mosquito biting pattern was variable among river basins, communities and biting location. Most of the outdoor bites occurred before 23:00 h, with a peak around 9:00 h, except SAL that had a bimodal pattern (19:00 and 22:00 h). Indoor biting also differed among sites with unimodal (SAL and LIB) and bimodal (VIB and URC) patterns. The *Plasmodium* infection rate was higher in mosquito populations from the Mazán River (VIB, LIB) compared to the Napo River in specimens collected inside and outside. The EIR was also higher in VIB and LIB, with higher values outdoors than indoors and within a range comparable to previous studies in the area [6, 48]. This reinforces the idea that, at least in these two communities, malaria transmission occurs also inside the houses.

The combination of a low HBR and almost zero EIR in Salvador with a considerable number of malaria cases, raises the question of where and when the actual transmission is occurring. This study did not explicitly record all the population activities or collect mosquitoes other than in the peridomestic area or inside the houses. We believe that infective mosquito bites may take place in areas where people work or perform other activities such as bathing in creeks or social activities such as playing soccer or watching TV. Our statistical multivariate model supports this premise, estimating up to 56 times higher EIR in the peridomestic area than indoors; therefore, individuals who expend more time outdoors during malaria vector peak activity constitute a high-risk group and specific mosquito feeding deterrent measures might be considered. In line with this, a multivariate analysis detected higher *P. vivax* prevalence related to occupation (loggers, fishermen and farmers) from URC and Gamitanacocha (Mazán River), reinforcing the idea that some malaria transmission might occur at some distance from the village sites [3]. Spatiotemporal identification and quantification of the prevailing malaria transmission would provide new evidence to orient specific control measures for vulnerable or at high risk populations [51, 52].

Some heterogeneities in biting behavior might be explained by intrinsic characteristics of the mosquito populations, such as genetic differentiation as a result of changed biting behavior. Two genetically distinct subpopulations of *Ny. darlingi* have been identified within the Iquitos area associated with habitat ecological characteristics (riverine *vs* highway) [48]. These subpopulations also presented variation in biting activity time and HBR estimates. In contrast, regarding biting behavior (exo-endophagic and biting time), *Ny. darlingi* appears to constitute a genetically homogeneous population [7]. Genetic characterization of the sampled mosquito populations was beyond the aim of our study and was not performed but further analysis might provide insightful information.

Our findings support the efficacy of the barrier screen methodology to intercept mosquito specimens suitable for blood meal identification analysis in this region [8]. The higher species richness composition detected with barrier screens compared with HLC suggests that other Anophelinae aside from *Ny. darlingi* are present at the time of the survey that were not collected using human baits. In summary, HBI was higher along the Mazán River than the Napo River, with VIB demonstrating the highest and SAL the lowest, with values similar to those described previously in the Peruvian Amazon and for *Ny. darlingi* [8]. In agreement with Moreno et al. [8], Galliformes, of non-human hosts, were the most common mosquito choice except in SAL, where cows were preferred (although in SAL, a few mosquitoes were collected by HCL or barrier screens). Additionally, blood meal sources were more diverse in URC and LIB than in the other two communities. In a longitudinal study in the area, bivariate models for *P. vivax* parasitemia identified a higher *P. vivax* prevalence associated with livestock in dwellings in URC [3]. In our study, the forage ratio estimation (after biomass adjustment) in this site detected cows as the second most common host, after Galliformes followed by humans. Therefore, heterogeneities in blood meal mosquito preferences and/or host availability may have an impact on the complexity of malaria transmission patterns in the area.

An association between housing structures (wall material) was detected for *P. falciparum* cases but not for *P. vivax*. In contrast, higher *P. vivax* parasite prevalence was recorded in houses with walls made of palm leaf or straw in Gamitanacocha (Mazán River) [3]. Even though most of the mosquitoes bit outdoors, we detected infected mosquitoes collected inside the dwellings in the Mazán River sites; therefore, housing improvements might be recommended in addition to the current vector control strategies [53, 54].

## Conclusions

Our research revealed heterogeneity in malaria transmission and vector bionomics at a microgeographical scale and that the peridomestic area in these communities contributes substantially to maintain *Plasmodium* transmission in the Peruvian Amazon. Even with elevated coverage and use of LLINs there is a gap in malaria control that needs to be addressed in the current elimination era. Specific strategies based on behavioral aspects of Neotropical malaria vectors and the human population should be developed and tested for their efficacy in these settings.

## Additional file

**Additional file 1: Table S1.** Animal host census in SAL, URC, LIB and VIB, Mazan district, Peru, performed in November 2016. **Table S2.** Summary of Anophelinae specimens collected indoors/outdoors (peridomestic). **Table S3.** Forage ratio (*w*_*i*_) and host selection index (*B*_*i*_) of *Ny*. *darlingi* in Mazán District sites between 2016 and 2017. **Figure S1.** Number of Anophelinae mosquitoes collected with barrier screen by position in Salvador (SAL), Urcomiraño (URC), Libertad (LIB) and Visto Bueno (VIB). *Abbreviations*: V-F, village-forest; V-BS, village-breeding site.

## Data Availability

The data supporting the conclusions of this article are included within the article. The raw data used and/or analyzed in this study are available from the corresponding author upon reasonable request.

## Abbreviations

EIR: entomological inoculation rate
GLMM: generalized linear mixed effect model
HBI: human blood index
HBR: human-biting rate
HLC: human landing catch
LIB: Libertad village
SAL: Salvador village
URC: Urco Miraño village
VIB: Visto Bueno village
