# Supplementary material for: Higher risk of malaria transmission outdoors than indoors by Nyssorhynchus darlingi in riverine communities in the Peruvian Amazon
Source: Parasit Vectors. 2019 Jul 29;12:374. doi: 10.1186/s13071-019-3619-0 (PMC6664538; doi:10.1186/s13071-019-3619-0)
Supplement: Supplementary file 1 — Additional file 1: Table S1. Animal host census in SAL, URC, LIB and VIB, Mazan district, Peru, performed in November 2016. Table S2. Summary of Anophelinae specimens collected indoors/outdoors (peridomestic). Table S3. Forage ratio (wi) and host selection index (Bi) of Ny. darlingi in Mazán District sites between 2016 and 2017. Figure S1. Number of Anophelinae mosquitoes collected with barrier screen by position in Salvador (SAL), Urcomiraño (URC), Libertad (LIB) and Visto Bueno (VIB). Abbreviations: V-F, village-forest; V-BS, village-breeding site. [file 13071_2019_3619_MOESM1_ESM.docx]

**Additional file 1**

**Higher risk of malaria transmission outdoors than indoors by *Nyssorhynchus darlingi* in the Mazán District in the Peruvian Amazon**

**Marlon P. Saavedra, Jan E. Conn, Freddy Alava, Gabriel Carrasco, Catharine Prussing, Sara A. Bickersmith, Jorge Sangama, Carlos Fernandez-Miñope, Mitchel Guzman, Carlos Tong, Carlos Valderrama, Joseph Vinetz, Dionicia Gamboa, Marta Moreno**

| **Table S1.** Animal host census in SAL, URC, LIB and VIB, Mazan district, Peru, performed in November 2016. | | | | | | | |
| --- | --- | --- | --- | --- | --- | --- | --- |
|  | **Host** | | | | | | |
| **Site** | **Chicken** | **Dog** | **Cat** | **Duck** | **Cow** | **Pig** | **Monkey** |
| SAL | 967 | 38 | 17 | 188 | 22 | 8 | 0 |
| URC | 333 | 92 | 18 | 80 | 7 | 31 | 0 |
| LIB | 783 | 77 | 29 | 72 | 23 | 25 | 4 |
| VIB | 191 | 10 | 14 | 51 | 4 | 16 | 0 |

| **Table S2.** Summary of Anophelinae specimens collected indoors/outdoors (peridomestic). | | | | | | | | |
| --- | --- | --- | --- | --- | --- | --- | --- | --- |
|  | **Site** | | | | | | | |
|  | **SAL** | | **URC** | | **LIB** | | **VIB** | |
| **Date** | **In** | **Out** | **In** | **Out** | **In** | **Out** | **In** | **Out** |
| March | 1 | 4 | 5 | 12 | 136 | 133 | 119 | 270 |
| June | 33 | 33 | 149 | 476 | 263 | 1179 | 457 | 2273 |
| September | 2 | 0 | 3 | 16 | 17 | 56 | 18 | 124 |
| November | 0 | 0 | 0 | 0 | 0 | 4 | 1 | 3 |
| March | 0 | 0 | 7 | 11 | 77 | 218 | 53 | 212 |
| **Total (%)** | 36 (49.3) | 37 (50.7) | 164 (24.2) | 515 (75.8) | 493 (23.7) | 1590 (76.3) | 648 (18.4) | 2882 (81.6) |

| **Table S3.** Forage ratio (*w_i_*) and host selection index (*B_i_*) of *Ny*. *darlingi* in Mazán District sites between 2016 -2017. | | | | | |
| --- | --- | --- | --- | --- | --- |
| **Host** | **Blood meal ID (*n*)** | **Host Abundance (*n*)** | **¹Forage Ratio A (*wi*)** | **Selection index (*Bi*)** | **²Forage Ratio B (*wi*)** |
| **SAL** |  |  |  | | |
| Human | 10 | 188 | 0.53 | 0.06 | 1.21 |
| Chicken | 0 | 0 | 0 | 0 | 0 |
| Cow | 4 | 22 | 1.94 | 0.23 | 6.73 |
| Pig | 3 | 8 | 3.56 | 0.43 | 2.99 |
| Dog | 4 | 38 | 2.25 | 0.27 | 0.59 |
| **URC** |  |  |  | | |
| Human | 88 | 164 | 1.78 | 0.09 | 0.78 |
| Chicken | 35 | 333 | 0.52 | 0.01 | 9.93 |
| Cow | 25 | 7 | 17.63 | 0.02 | 2.02 |
| Pig | 5 | 31 | 1.8 | 0.04 | 0.25 |
| Dog | 4 | 92 | 0.16 | 0.84 | 0.18 |
| **LIB** |  |  |  | | |
| Human | 77 | 167 | 3.35 | 0.24 | 1.05 |
| Chicken | 34 | 783 | 0.32 | 0.03 | 4.42 |
| Cow | 27 | 23 | 8.21 | 0.02 | 0.67 |
| Pig | 6 | 25 | 1.74 | 0.12 | 0.31 |
| Dog | 3 | 77 | 0.38 | 0.59 | 0.4 |
| **VIB** |  |  |  | | |
| Human | 320 | 35 | 4.76 | 0.8 | 1.5 |
| Chicken | 126 | 191 | 0.44 | 0.03 | 6.08 |
| Cow | 3 | 4 | 0.5 | 0.07 | 0.16 |
| Pig | 1 | 16 | 0.04 | 0.01 | 0.04 |
| Dog | 3 | 10 | 0.2 | 0.08 | 0.01 |
| ^1^Forage Ratio A was calculated using the number of hosts. ^2^Forage Ratio B was calculated using the biomass of hosts. Mean weight of hosts was: human (65 kg), dog (25kg), chicken (1.5kg), pig (90kg), cow (250 kg). | | | | |  |


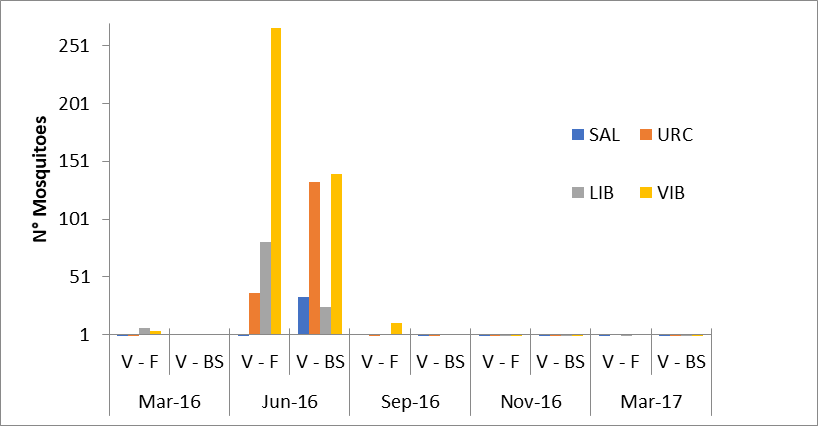


**Figure S1.** Number of Anophelinae mosquitoes collected with Barrier Screen by position in Salvador (SAL), Urcomiraño (URC), Libertad (LIB) and Visto Bueno (VIB). V-F: village-forest; V-BS: village-breeding site.
